# From Clinical Perception to Implicit Bias: Understanding Personality Traits in Lymphoma Patients

**DOI:** 10.3390/cancers17111743

**Published:** 2025-05-22

**Authors:** Fátima Roso-Bas, María Dolores Alonso-Llobregat, Leyre Bento, Blanca Sánchez-González, Layla Aoukhiyad Lebrahimi, Inés Herráez Balanzat, Pilar García-Dilla, Francesc García-Pallarols, Sara Nistal Gil, Samuel Romero, María-Jesús Vidal, Carolina De Bonis-Braun, Yapci Ramos de León, María Stefania Infante, Eva Domingo-Domenech, Susana Ramírez, Joan Bargay, Antonia Sampol, Antonio Salar, Antonio Gutiérrez

**Affiliations:** 1 Clinical Practice and Biology of the Hematological Malignancies Research Group, IdISBa, Son Espases University Hospital, 07120 Palma de Mallorca, Spain; fatima.roso@ibsalut.es (F.R.-B.); mariad.alonsol@ibsalut.es (M.D.A.-L.); leyre.bento@ssib.es (L.B.); ines.herraez@hsll.es (I.H.B.); jbargay@hsll.es (J.B.);; 2 General Directorate for Mental Health of the Ministry of Health of the Balearic Islands, 07010 Palma de Mallorca, Spain; 3 Department of Quality and Patient Safety, Health Care Directorate of the Balearic Islands Health Service, 07003 Palma de Mallorca, Spain; layla.aoukhiyad@ibsalut.es; 4 Unit of Lymphoma, Department of Hematology, Son Espases University Hospital, 07120 Palma de Mallorca, Spain; 5 Department of Hematology, Hospital del Mar, 08003 Barcelona, Spain; bsanchezgonzalez@psmar.cat (B.S.-G.); mgarciad@psmar.cat (P.G.-D.); fgpallarols@gmail.com (F.G.-P.); asalar@parcdesalutmar.cat (A.S.); 6 IMIM, Hospital del Mar Research Institute, 08003 Barcelona, Spain; 7 Department of Hematology, Son Llatzer University Hospital, 07198 Palma de Mallorca, Spain; 8 Hospital HLA Universitario Moncloa, 28008 Madrid, Spain; sara_nistal@gmail.es; 9 Department of Hematology, University and Polytechnic La Fe Hospital, 46026 Valencia, Spain; romero_sam@gva.es; 10 Department of Hematology, Hospital Universitario de León, 24071 León, Spain; mjvidalm2010@gmail.com; 11 Hematology, Hospital Universitario De Canarias, 38320 La Laguna, Spain; cbonisbraun@gmail.com; 12 Department of Hematology, Hospital Dr José Molina Orosa, 35550 Lanzarote, Spain; yapcirdl@hotmail.com; 13 Department of Hematology, University Hospital Infanta Leonor, 28031 Madrid, Spain; ms.infante@gmail.com; 14 Department of Hematology, Institut Catala d’Oncologia, Hospital Duran i Reynals, IDIBELL, 08908 L’Hospitalet de llobregat, Spain; edomingo@iconcologia.net; 15 Department of Hematology, University Hospital Juan Ramón Jiménez, 21005 Huelva, Spain; susana84algamitas@hotmail.com

**Keywords:** personality assessment, Five-Factor Model, psycho-oncology, lymphoma, Hodgkin’s disease, ageism

## Abstract

**Background/Objectives**: Some haematologists share the perception that patients with Hodgkin’s lymphoma (HL) behave and manifest emotional expressions in a characteristic way. Previous research suggested a unique personality profile in HL patients compared to the general population. This study aimed to analyse and compare the personality traits of HL and NHL patients to identify potential differences. **Methods:** In this cross-sectional, descriptive, multicentre and replicative study, we included patients with HL and NHL from the Spanish Group of Lymphoma (GELTAMO). Personality traits and other psychosocial variables were compared between these two groups and the reference population. We used a semi-structured interview to collect demographic and psychosocial variables, and the NEO Five-Factor Inventory to assess personality traits. **Results:** Our findings indicate that HL and NHL patients share similar personality profiles, suggesting that the perceived differences do not stem from personality factors. Significant differences were only observed in age (HL > NHL; age: *p* = 0.003). These results led us to propose a new explanatory hypothesis centred on ageism. **Conclusions:** Our results confirm that the personality profiles of patients with any type of lymphoma are consistent with each other and with those found in the broader cancer patient population, indicating that differences observed by clinicians might be due to perceptual biases. Age, as a differentiating factor between these patient groups, suggests ageism as a potential underlying cause of these biases. Further research is required to explore the clinical implications of such stereotypical perceptions among patients that could ultimately lead to issues with patient–provider relationships and patient safety.

## Simple Summary

This study investigates the differences in personality traits between patients with Hodgkin’s lymphoma (HL) and non-Hodgkin’s lymphoma (NHL), in response to the perception of some haematologists regarding distinctive emotional behaviours in HL patients. Through interviews and the NEO Five-Factor Inventory, personality characteristics and psychosocial variables of both groups were compared. The results indicate that HL and NHL share similar personality profiles, with significant differences only in age, with HL patients being younger. This suggests that the differences observed by clinicians may be due to perceptual biases related to age, a phenomenon known as ageism.

## 1. Introduction

Growing interest exists in exploring the associations between personality traits and the development and maintenance of physical and psychological illnesses [1]. Research consistently supports the Five-Factor Model (FFM) as a robust framework for understanding individual differences [2,3,4], which are viewed as psychological characteristics with concrete manifestations influencing well-being and health outcomes [5,6].

Research links low neuroticism and high conscientiousness with better health behaviours and outcomes [7], suggesting that these traits act as protective factors, particularly for mental health in cancer patients [8] and as indicators of disease adaptation [9]. Conversely, high neuroticism and/or low conscientiousness are associated with elevated inflammatory markers and increased chronic disease risk [10,11]. The early identification of these personality characteristics is crucial for preventive medicine influencing both mental and physical health [12,13] and aiding psychosocial adjustment to cancer [14].

While much research has explored personality in solid-tumour patients [1,7,15,16], fewer studies focus on haematological cancers [17,18,19]. Understanding personality in these patients is crucial as it can predict treatment adherence and self-care engagement [3,20].

Lymphomas are haematological malignancies originating from B, T, or NK cells. Their treatment typically includes forms of chemotherapy or immunochemotherapy, sometimes combined with radiotherapy. With these approaches, many lymphomas are potentially curable. In particular, Hodgkin’s lymphoma (HL) is highly chemosensitive and primarily affects younger individuals, showing a bimodal age distribution: approximately 50–60% of patients are under 40 years old, while 30% are older than 60. In contrast, most Non-Hodgkin’s lymphomas (NHLs) occur in people of older age and are mainly treated with immunochemotherapy.

Anecdotal observations by haematologists suggest potential individual differences in interaction, emotional expression, and self-regulation between patients with HL and NHL. A previous study [19] identified a distinct personality profile in HL patients post-treatment, characterised by high neuroticism and low scores in extraversion, agreeableness, and responsibility, contrasting with the general population.

This exploratory study aims to verify whether this profile is unique to HL or also present in other types of lymphoma (NHL), thereby addressing whether personality differences correspond to lymphoma type. Additionally, this research extends the analysis to demographic and psychological variables, exploring their relationship with lymphoma diagnosis to provide a more comprehensive understanding of these associations.

## 2. Materials and Methods

### 2.1. Aims and Design of the Study

Building on the results of our previous study, which found that patients with HL differed from the general population in their personality profile, we used the same assessment tools to analyse a cohort of patients with NHL. The primary aim of this study was to compare the personality traits between a cohort of patients with HL and a cohort of those with NHL. Additionally, similar to the previous study, the personality profiles of these two groups were compared with the personality profiles of the reference population (national scale of personality of the general population). This research is a replicative, exploratory, descriptive, cross-sectional, and multicentre study.

### 2.2. Participants/Sample

**Figure 1:**
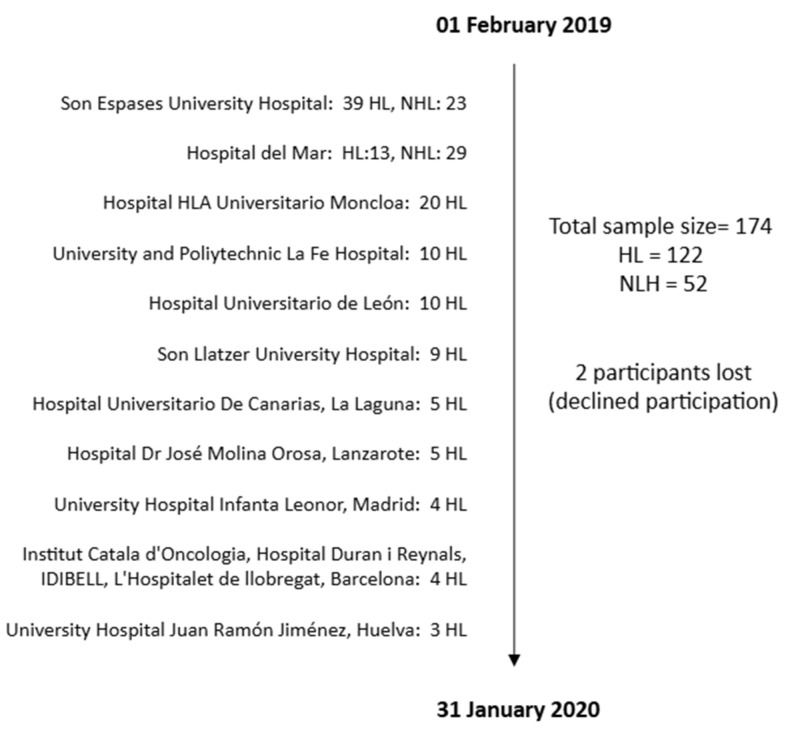
Patient recruitment process for the study.

This study was conducted by the Spanish Group of Lymphoma (GELTAMO), the leading cooperative group in Spain composed of haematologists specialised in the treatment of lymphoma. Newly diagnosed HL patients were recruited across a multicentre network of 11 GELTAMO hospitals. Newly diagnosed NHL patients, however, were recruited from the two hospitals that were central to the initiation of this study (an overview of the patient recruitment process is presented in Figure 1). This work included adult patients aged 18 to 75 years, newly diagnosed with HL (cases) or other aggressive lymphomas (controls), and prior to treatment initiation. The exclusion criteria were language barriers and cognitive or neurological impairments that could interfere with the ability to provide informed consent or complete the study procedures.

We employed consecutive sampling, a non-probability sampling technique in which all HL and NHL patients diagnosed over 12 months (1 February 2019 to 31 January 2020) in the abovementioned centres were selected according to the criteria set forth by the International Classification of Diseases [21]. This method was deemed appropriate for our research as it significantly reduces selection bias (compared to other non-probability techniques) and yields a more representative sample of the clinical population of interest within the hospital setting [22]. A minimum target of 50 patients was established for the NHL group to ensure adequate statistical power for comparisons between the two substantial cohorts, considering the overall study sample size.

### 2.3. Procedure

**Figure 2:**
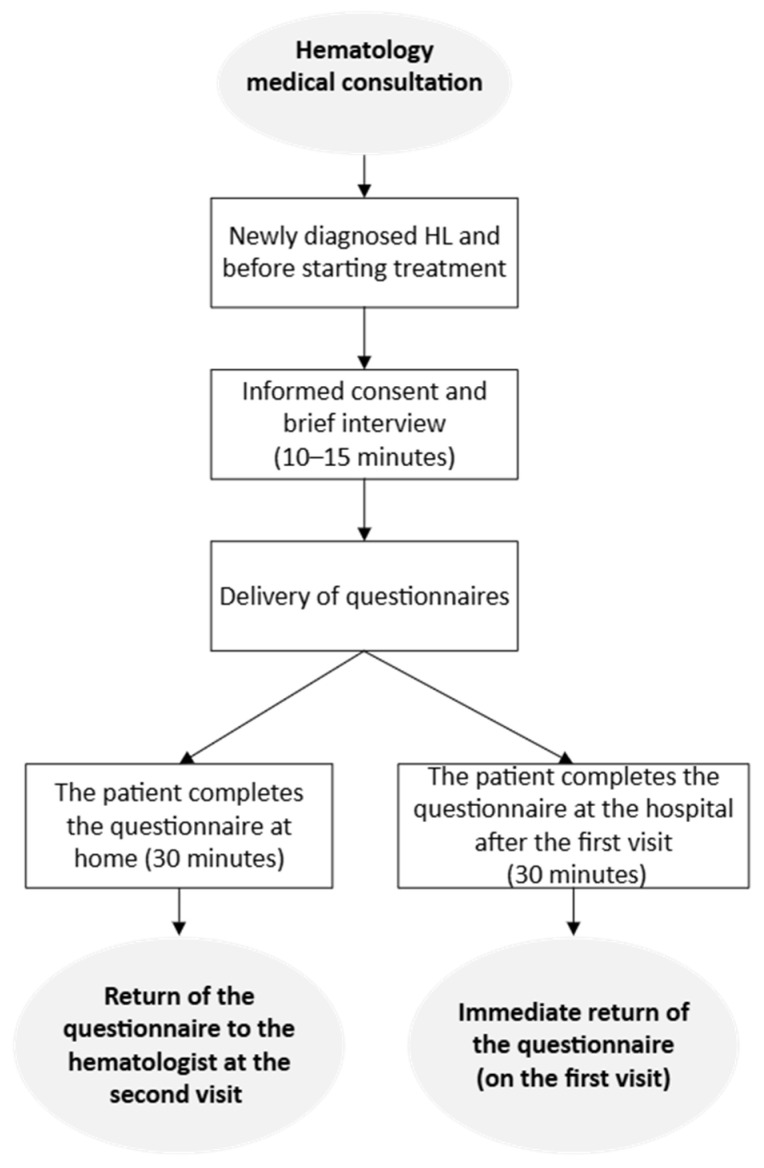
Flowchart of the data collection process.

To mitigate selection bias, uniform instructions (see Supplementary Materials) were adhered to across each participating hospital. All eligible patients were invited to participate in the study. Upon agreeing, participants signed an informed consent form before inclusion. A haematologist then conducted a brief 15 min interview to gather sociodemographic and psychosocial data. After the interview, patients were provided with an instruction sheet (see Supplementary Materials) and questionnaires, which they completed before starting chemotherapy, taking approximately 30 min (see the flowchart in Figure 2).

The patient profiles derived from this study were compared with those of HL patients from previous work [19]. In the referenced study, 96 HL survivors treated between January 2007 and December 2017 were examined. Patients’ contact details were extracted from the Pharmacy and Haematology databases of two hospitals in the Balearic Islands (Son Espases University Hospital and Son Llatzer University Hospital) and one hospital in Catalonia (Hospital del Mar). The selection process mirrored that of the prior referenced study. Patients were contacted by phone and invited to sign the informed consent form, participate in the interview, and complete the questionnaires.

### 2.4. Measures

A semi-structured questionnaire of 12 questions was administered to collect data on sociodemographic variables (age, gender, civil status, educational level, and cohabitation) and psychological variables (perception of social support, stressful life events, impact of family cancer history, need for psychological support, psychiatric diagnosis, and history of autolytic thoughts and suicide attempts).

#### NEO Five-Factor Inventory (NEO-FFI)

The NEO-FFI is a 60-item measure developed within the framework of the Five-Factor Model (FFM) by Costa and McCrae and adapted for the Spanish population [23]. A five-point Likert scale of agreement was employed, ranging from 1 (strongly disagree) to 5 (strongly agree). General (i.e., non-pathological) personality can be organised into five dimensions: Neuroticism (reflecting the propensity to experience negative emotions such as anxiety and depression); Extraversion (indicating the tendency to be sociable, assertive, warm, cheerful, and energetic); Openness to Experience (denoting the inclination to be imaginative, unconventional, and sensitive to emotional, and artistic experiences); Agreeableness (representing the tendency to be trusting, modest, altruistic, and cooperative); and Conscientiousness (characterising an individual’s inclination to be persistent, organised, dependable, and rule-abiding).

### 2.5. Statistical Analysis

Descriptive data are presented as frequencies and percentages. To assess associations between sociodemographic and psychosocial variables, we utilised the Mann–Whitney U test, Fisher’s exact test, and Monte Carlo test as appropriate. Personality traits scores from our samples were compared with a national reference sample using the one-sample Wilcoxon signed-rank test. For comparisons of personality trait scores between the two independent samples (HL and NHL), the Mann–Whitney U test was employed. The reliability of the NEO Five-Factor Inventory scales was evaluated using Cronbach’s alpha.

Statistical significance was set at *p* < 0.05, and all tests were two-sided. Data analysis was performed using IBM SPSS Statistics version 19 for PC (IBM Corporation, Armonk, NY, USA).

## 3. Results

### 3.1. Sociodemographic and Psychosocial Data

Table 1 displays the sociodemographic characteristics of both study cohorts. There are significant age differences between the groups (*p* = 0.003), with age of the HL group being lower (median = 39, range = 70) compared to the NHL group (median = 51, range = 58). Gender composition differed between the groups; however, this variation did not reach statistical significance: women were the majority in the HL group (54.9%), whereas men were more common in the NHL group (59.6%). No significant differences were observed between the cohorts in terms of civil status, education level, or living arrangements. Regarding the psychosocial variables (Table 2), no significant differences were found.

### 3.2. Personality Trait Contrasts

As detailed in Table 3, our comparison of personality traits between the HL and NHL groups revealed no significant differences.

In our study, we compared the personality traits of survivors from our previous study [19] with those of newly diagnosed HL patients. The results, detailed in the Supplementary Materials (Table S1), show very similar profiles across all examined traits, indicating no significant differences.

Further contrasts were made between patients with HL and NHL with respect to the personality traits against a normative reference population (Table 4). It should be noted that, in the HL group, all traits significantly differed from the normative population. In the NHL group, significant differences to the norm were observed in neuroticism and agreeableness, with a trend towards lower conscientiousness.

Additionally, we analysed the frequency and percentage of subjects displaying high neuroticism and low scores in extraversion, openness, and agreeableness, and based on their diagnosis. No significant differences were found in these distributions.

## 4. Discussion

Given the positive results of the initial study [19], we anticipated identifying a differential personality profile in patients with HL compared to those with NHL and the general population. However, our results also revealed differences between the NHL and the general population, but no significant differences between NHL and HL. Age emerged as the only differentiating factor among the samples; therefore, the hypothesis of personality trait differences between HL and NHL patients is not supported. This finding led us to shift our analytical focus from patients’ personality profiles to factors related to physicians. We have proposed a new explanatory hypothesis based on the only consistent difference observed: age. This hypothesis—which, to our knowledge, is introduced for the first time in the present manuscript—suggests that perception biases, including ageism, may influence the physician–patient relationship.

The current study aims to elucidate why many physicians perceive differences in behavioural and emotional responses between HL and other types of lymphoma. Our findings reveal significant variations in some personality traits between HL and NHL patients and the general reference populations. However, the personality profiles within our HL and NHL samples showed remarkable similarity, with no differences even when compared with the cohort of HL survivors or profiles cited in other oncology studies [19]. This suggests that while personality traits may play a role in the development of cancer, they do not necessarily explain the observed behavioural differences between HL and NHL patients reported by treating physicians.

In general, we can see that people diagnosed with lymphoma share a specific profile that contrasts with the reference population without cancer. Both HL and NHL exhibit similar personality patterns, which are high scores in neuroticism and low scores in extraversion, agreeableness, and conscientiousness. These profiles are similar to those defined as “overcontrolled” in community samples [6] or a “distressed personality class” in cancer patients undergoing chemotherapy [24,25]. Conversely, these patterns are the opposite to what has been describe as a factor of general adaptivity composed of low neuroticism and high extraversion, agreeableness, and responsibility associated with better therapeutic goals in mental health therapies [26] and with high levels of psychological well-being [27]. It is widely known that people who score high in neuroticism tend to experience life events, cancer treatment, and follow-up as more threatening and stressful [8], which is linked to worse mental health outcomes in cancer [28]. Particularly in patients with lymphoma, it has been found that neuroticism was the personality trait most related to psychological distress [18].

We highlight that identical personality patterns emerged both in survivors after receiving chemotherapy and in newly diagnosed patients who had not yet started it. Many researchers expect personality changes after stressful events [29,30]; however, the present work suggests that personality traits in these patients are independent of treatment, thus supporting the hypothesis of stability against major stressors. Nevertheless, several studies aiming to determine the influence of acute life stressors such cancer and chemotherapy on related personality or dispositional variables has led to mixed results [16,31].

Regarding psychosocial factors, significant associations were found between lymphoma diagnosis and age. As expected, there were younger patients in the HL group [32]. It should be considered that as people age, the neuroticism trait decreases significantly as neuroticism moderates with age [31]. For this reason, since many HL patients are younger than NHL patients, higher neuroticism scores would be expected in the HL group. However, as stated, this difference did not appear in our samples. This would probably indicate that the high neuroticism trait, often seen in cancer patients, may override age-related variations with ageing.

In our samples, both HL and NHL patients exhibited high levels of neuroticism and lower scores in extraversion, agreeableness, and conscientiousness. If clinicians attribute traits such as irritability, impulsiveness, and lack of self-control specifically to the HL group, this may reflect age-related perceptual biases, a phenomenon known as ageism.

This concept involves how groups or individuals perceive stereotypes, express prejudices, and act discriminatively towards others based on age [33,34], with these biases potentially being positive or negative [35] and explicit or implicit [36].

These automatic biases, even if not consciously held, can lead to specific expectations about entire groups of people, or patients in this case [37]. The lack of confirmed personality differences suggests that pre-existing, potentially stereotypical beliefs about HL and NHL patients might have influenced physician expectations, rather than actual differences in personality.

Our study suggests that negative ageism may influence clinicians’ perception of younger patients, particularly given findings from the European Social Survey (ESS) that younger people experience the highest levels of age discrimination [38,39]. Francioli and North [40] analysed stereotypes and biases toward young adults, and the interesting aspect of this research lies in the fact that, unlike ageism toward older adults, this bias emerges from derogatory comparisons between generational cohorts, with young adults being subject to more severe social judgements than older adults. However, there is scarce literature regarding ageism against younger populations, specifically in the healthcare and social care sectors [40,41]. Conversely, older patients might receive more permissive treatment under similar circumstances, a pattern supported by literature indicating a general positive attitude towards older patients in healthcare settings [42,43], although there are inconsistencies regarding this topic [44].

Several limitations of our study warrant acknowledgment. The NHL sample was smaller and more heterogeneous compared to the HL group. This variability in clinical characteristics and sample size may affect the robustness of the comparisons and the ecological validity and generalisability of our findings.

Although the NEO-FFI-R demonstrates high reliability in measuring basic personality domains and enhances study feasibility through its brevity, it fails to capture detailed facet-level information, thus limiting a more nuanced exploration of these behaviours.

Finally, despite the anonymous nature of the self-administered questionnaires, the potential influence of social desirability bias cannot be dismissed, as participants may have perceived evaluation by healthcare professionals, potentially affecting their responses [45].

Future research would benefit from a mixed-methods, longitudinal approach that includes a larger and more homogeneous NHL sample. These methodological refinements could lead to more accurate, nuanced results and a deeper understanding.

Further studies are needed to explore how healthcare professionals’ perceptions vary with the age of lymphoma patients to better understand these biases and their implications.

## 5. Conclusions

Our findings did not confirm anticipated differences in personality traits between Hodgkin’s lymphoma (HL) and Non-Hodgkin’s lymphoma (NHL) patient groups, necessitating an examination of social perceptions shaped by implicit biases. Notably, age emerged as the primary distinguishing factor between the groups in our study, leading us to propose ageism as a potentially significant underlying influence.

Ageism, closely linked to stereotypy, is increasingly recognised in healthcare for its clinical implications, contributing to biased clinical decisions and potentially harming patient safety through the overuse or underuse of treatments [46].

The most immediate implication of these results is the critical need to raise awareness among healthcare professionals regarding the potential for unconscious biases to affect their perceptions of HL and NHL patients. Recognising these biases can encourage more mindful communication and interaction, fostering more equitable and trusting patient–physician relationships.

Consequently, patients experiencing fair and unbiased treatment may exhibit greater engagement in their care, improved adherence, and better outcomes. These findings strongly support the integration of implicit bias and ageism training into medical education and continuing professional development curricula.

## Figures and Tables

**Table 1:** Sociodemographic descriptive data and sample analysis.

	HL	NHL	
Age	Med (Rank)	Med (Rank)	*p*
	39 (70)	51 (58)	0.003 *^a^
Gender	n (%)	n (%)	*p*
Male	55 (45.1)	31 (59.6)	0.098 ^b^
Female	67 (54.9)	21 (40.4)	
Civil Status			
Single	59 (48.8)	16 (30.8)	0.066 ^c^
Married	48 (39.7)	31 (59.6)	
Widowed	3 (2.5)	-	
Separated/Divorced	11 (9.1)	5 (9.6)	
Educational levels			
Uneducated	1 (0.8)	-	0.197 ^c^
Primary	25 (20.7)	12 (23.1)	
Secondary	39 (32.2)	24 (46.2)	
High School	56 (46.3)	16 (30.8)	
Cohabitation			
Alone	14 (11.7)	8 (15.4)	0.841 ^c^
With relatives	103 (85.8)	43 (82.7)	
Other	3 (2.5)	1 (1.9)	

* Statistically significant *p*-value < 0.05. ^a^ Mann–Whitney U test. ^b^ Fisher’s exact test. ^c^ Monte Carlo simulation (number of simulations = 10.000).

**Table 2:** Psychosocial descriptive data and sample analysis.

	HL	NHL	
	n (%)	n (%)	*p*
Perception of social support			
No social support	1 (1)	1 (2.6)	0.296 ^a^
Moderate social support	13 (13)	2 (5.3)	
High social support	86 (86)	35 (92.1)	
Stressful life events			
No life stressor	42 (35)	14 (26.9)	0.654 ^a^
Mild life stressor	14 (11.7)	9 (17.3)	
Moderate life stressor	21 (17.5)	10 (19.2)	
Intense life stressor	43 (35.8)	19 (36.5)	
Impact of family cancer history			
No	30 (25)	10 (19.2)	0.217 ^a^
Mild impact	28 (23.3)	17 (32.7)	
Moderate impact	32 (36.7)	8 (15.4)	
Intense impact	30 (25)	17 (32.7)	
Need for psychological support			
No	87 (79.1)	33 (64.7)	0.368 ^b^
Yes	34 (28.1)	18 (35.3)	
Psychiatric diagnosis			
No	105 (86.8)	46 (88.5)	1.0 ^b^
Yes	16 (13.2)	6 (11.5)	
History of autolytic thoughts			
No	97 (84.3)	45 (86.5)	0.817 ^b^
Yes	18 (17.7)	7 (13.5)	
History of suicide attempts			
No	112 (98.2)	39 (95.1)	0.285 ^b^
Yes	2 (1.8)	2 (4.9)	

^a^ Monte Carlo simulation (number of simulations = 10.000). ^b^ Fisher’s exact test.

**Table 3:** Contrasts between the newly diagnosed HL and NHL groups.

NEO-FFI	α	HL (n = 122)		NHL (n = 52)			
		M (SD)	Med (Rank)	M (SD)	Med (Rank)	*U*	*p*
Neuroticism	0.827	19.89 (8.39)	19 (37)	19.28 (8.08)	17 (39)	2934.5	0.651
Extraversion	0.837	30 (7.34)	30 (34)	30.33 (8.52)	31 (40)	2834.5	0.496
Openness	0.807	26.64 (7.72)	26 (39)	27.53 (8.37)	26.5 (37)	2787.0	0.341
Agreeableness	0.722	31.47 (5.80)	31 (26)	30.33 (7.53)	31 (32)	2918.5	0.693
Conscientiousness	0.817	32.57 (7.13)	33 (37)	34.07 (7.30)	34.5 (33)	2627.5	0.117

**Table 4:** Contrasts of the HL and NHL groups with respect to the normative values of people without cancer and proportion of patients with scores above/below cut score.

NEO-FFI	Reference Population		HL Sample (n = 122)		NHL Sample (n = 52)	
	Mean (SD)	Med	Mean (SD)	Med (Rank)	Mean (SD)	Med (Rank)
Neuroticism	15.35 (7.40)	14	19.89 (8.39)	19 (37)	19.28 (8.08)	17 (39)
			*p* = 0.000 *		*p* = 0.000 *	
	High cut score = 18		55.1%		46.2%	
	*X*^2^ = 0.82; *p* = 0.364 ^a^					
Extraversion	32.59 (6.35)	33	30 (7.34)	30 (34)	30.33 (8.52)	31 (40)
			*p* = 0.000 *		*p* = 0.057	
	Low cut score = 28		44.5%		31.4%	
	*X*^2^ = 2.04; *p* = 0.152 ^a^					
Openness	28.64 (6.56)	29	26.64 (7.72)	26 (39)	27.53 (8.37)	26.5 (37)
			*p* = 0.001 *		*p* = 0.293	
	Low cut score = 24		40.7%		32.7%	
	*X*^2^ = 0.66; *p* = 0.415 ^a^					
Agreeableness	32.79 (5.67)	33	31.47 (5.80)	31 (26)	30.33 (7.53)	31 (32)
			*p* = 0.009 *		*p* = 0.034 *	
	Low cut score = 29		37.8%		41.2%	
	*X*^2^ = 0.05; *p* = 0.810 ^a^					
Conscientiousness	36.01 (6.02)	36	32.57 (7.13)	33 (37)	34.07 (7.30)	34.5 (33)
			*p* = 0.000 *		*p* = 0.176	
	Low cut score = 32		48.7%		32.7%	
	*X*^2^ = 3.16; *p* = 0.075 ^a^					

Wilcoxon signed-rank test was used to contrast HL and NHL samples with reference population. * Statistically significant at *p*-value < 0.005. ^a^ Yates continuity correction.

## Data Availability

The data presented in this study are available upon reasonable and justified request from the corresponding author.

## Supplementary Materials

The following supporting information can be downloaded at https://www.mdpi.com/article/10.3390/cancers17111743/s1: Instructions provided to participating hospitals and patients; Table S1: Contrasts between the group of surviving HL patients and the group of newly diagnosed HL patients from the previous study [19].

## Abbreviations

The following abbreviations are used in this manuscript:

HL	Hodgkin’s Lymphoma
NHL	Non-Hodgkin’s Lymphoma
GELTAMO	Spanish Group of Lymphoma
FFM	Five-Factor Model
NEO-FFI	NEO Five-Factor Inventory
ESS	European Social Survey
