# Supplementary material for: From Clinical Perception to Implicit Bias: Understanding Personality Traits in Lymphoma Patients
Source: Cancers (Basel). 2025 May 22;17(11):1743. doi: 10.3390/cancers17111743 (PMC12153871; doi:10.3390/cancers17111743)
Supplement: Supplementary file 1 [file cancers-17-01743-s001.zip › cancers-3590551-supplementary.pdf]

Instructions for the Principal Investigators of Each Center

The present study aims to investigate the differential personality traits of patients with Hodgkin's lymphoma. It is a study with a very different work methodology from the one we are used to using in most onco-hematological studies. For this reason, it is very important that we are able to homogenise our work system between the various participating centers.

Among the limitations of this study is the risk of two possible biases:

- Selection bias: that we include only the most cooperative patients.

- Unreliable response bias: which can occur if the patient is accompanied by family, friends or healthcare professionals at the time of filling out the questionnaires. This bias can also occur if the patient believes that their answers will be public, will be known by their therapists or will be recorded in their medical history.

For all these reasons, it is very important that the participating hospitals commit to:

1) Include all patients diagnosed with Hodgkin lymphoma who meet the inclusion criteria during the recruitment period (February 2019 to January 31, 2020) to avoid potential selection bias.

2) That the patient completes the questionnaires without the presence of family, friends or health personnel. Only the initial 10–15-minute interview will be conducted by the investigator inviting the patient's companions to leave the consultation.

3) That the patient is insisted on the importance of their answers being truthful and that anonymity is guaranteed, since their answers will be entered into an anonymized database. So that doctors or health personnel cannot read the answers, the questionnaires will be placed in a sealed envelope at the end of them.

To anonymise patients, each patient must be assigned a unique identifier with the following data:

- Center Initials
- Correlative number of inclusion of the center
- Patient's initials
- Example: HUSE1AGCT, HUSE1CLLT

Instructions for Completing the Questionnaires. Patient Version

STUDY ON PERSONALITY TRAITS ASSOCIATED WITH LYMPHOMAS

After deciding to participate in this research and signing the informed consent document, you have conducted an interview with a member of the research team and have received a series of questionnaires to fill out calmly at home. Below, we suggest how to complete them so that the results are as valid as possible and you do not get tired during the completion of the task. Please read the following points carefully and try to follow them.

Thank you very much for your collaboration.

• Check that you have been given the following questionnaires. If you are missing any, please tell your haematologist:

1. NEOFFI (60 questions), questionnaire on personality traits.
2. STAI-E-R (40 questions), two questionnaires on anxiety
3. BDI (21 questions), questionnaire on mood.

• Whether you do it in the waiting room or at home, look for a time when you are relaxed and without physical discomfort.

• Try to answer alone without the help of family members.

• It is important to answer all the questions, check if the questionnaire continues on the back of the page.

• If you have doubts about any item, you can mark it and resolve your doubts on the next visit.

• When finished, gather all the questionnaires in the same envelope and seal it to protect your confidentiality.

• Remember to give the envelope to the hematologist immediately or at the next visit.

Table S1. Contrasts between the group of surviving HL patients and the group of newly diagnosed HL patients.

NEO-FFI	Survivors (n = 96) *	Newly Diagnosed (n = 122)				
	**Mean (SD)**	**Mean (SD)**	**Mean Differences**	**t**	**CI (95%)**	***p***
Neuroticism	20.48 (9.34)	19.89 (8.39)	0.59	0.47	(−1.84; 3.02)	0.633
Extraversion	30.28 (7.92)	30 (7.34)	0.28	0.26	(−1.82; 2.40)	0.789
Openness	28.61 (7.65)	26.64 (7.72)	1.97	1.78	(−0.20; 4.14)	0.075
Agreeableness	31.43 (6.34)	31.47 (5.80)	−0.04	−0.05	(−1.72; 1.62)	0.956
Conscientiousness	32.24 (7.32)	32.57 (7.13)	−0.32	−0.32	(−2.31; 1.65)	0.743

* LH sample from previous study (Roso-Bas et al., 2021)
